# Atezolizumab Plus Bevacizumab Versus Durvalumab Plus Tremelimumab for Advanced Hepatocellular Carcinoma: A Propensity-Score-Matched Analysis

**DOI:** 10.3390/biomedicines14071638

**Published:** 2026-07-21

**Authors:** Sarina Ailawadi, Sepideh Mehravar, Jennifer E. Murphy, Michael H. Storandt, Amit Mahipal

**Affiliations:** 1 Department of Medicine, University Hospitals, Case Western Reserve University, 11100 Euclid Ave, Cleveland, OH 44106, USA; sarina.ailawadi@uhhospitals.org; 2 Department of Medicine, Cedars-Sinai Medical Center, 8700 Beverly Blvd, Los Angeles, CA 90048, USA; sepideh.mehravar@cshs.org; 3 Clinical Research Center, University Hospitals, Case Western Reserve University, 11100 Euclid Ave, Cleveland, OH 44106, USA; jennifer.murphy@uhhospitals.org; 4 Department of Medical Oncology, Mayo Clinic, 200 First St. SW, Rochester, MN 55905, USA; storandt.michael@mayo.edu; 5 Department of Medical Oncology, University Hospitals Seidman Cancer Center, Case Western Reserve University, 11100 Euclid Ave, Cleveland, OH 44106, USA

**Keywords:** hepatocellular cancer, atezolizumab, bevacizumab, tremelimumab, durvalumab

## Abstract

**Background**: Atezolizumab plus bevacizumab (A + B) and durvalumab plus tremelimumab (D + T) are approved first-line systemic therapy options for advanced hepatocellular carcinoma (HCC), with no head-to-head comparison. In this propensity-matched analysis, we compared the survival of patients with advanced HCC who received either A + B or D + T therapy. **Methods**: Patients with advanced HCC who received A + B or D + T treatment in the first-line setting were identified using the TriNetX platform, a health research network that provides access to electronic health record data from 112 healthcare organizations. Propensity-score-matched (PSM) analysis was conducted, and the median overall survival (OS) was estimated using the Kaplan–Meier method. **Results**: We identified 2819 patients with HCC who were treated with A + B or D + T: 2031 patients received A + B and 788 received D + T. Compared to patients in the A + B cohort, those who received D + T were more likely to be older (median age: 68.4 vs. 66.9 years), have lower platelet counts (186.8 vs. 201.3), higher prevalence of hypoalbuminemia with levels < 2.7 g/dL (35.2% vs. 28.4%), higher prevalence of bilirubin levels between 2.0 and 2.9 mg/dL (28.8% vs. 23.8%), and decreased INR with levels between 0.0 and 1.6 (92.1% vs. 86.3%). After PSM, 1536 patients were included in the survival analyses, with all variables adequately matched. There was no significant difference in the median OS between those receiving A + B or D + T [16.3 vs. 22.5 months, hazard ratio (HR) 0.89, 95% confidence interval (CI) 0.76–1.0]. Higher rates of immune-mediated colitis were noted in the D + T group. **Conclusions**: Similar survival rates were observed among patients with advanced HCC who received A + B and D + T in the first-line setting. Our study suggests that both A + B and D + T are valid treatment options, and that therapy can be tailored based on comorbidities, adverse effects, and patient preferences.

## 1. Background

Primary liver cancer, of which hepatocellular carcinoma (HCC) comprises 75–85% of cases, is the sixth most common cancer globally and the third leading cause of cancer-related death [1,2]. The incidence of HCC in the United States (U.S.) is projected to increase in the coming years, which may be partly due to the increased incidence of metabolism-associated fatty liver disease [3,4]. Historically, sorafenib has been the only systemic therapy option for patients with advanced HCC in the first-line setting; however, the introduction of immunotherapy for HCC has changed this treatment paradigm [5,6].

Atezolizumab plus bevacizumab (A + B) was the first regimen to demonstrate improved overall survival (OS) when compared to sorafenib in the first-line setting, leading to FDA approval. The IMBrave150 phase III clinical trial reported a median overall survival (mOS) of 19.2 months compared to 13.4 months in the sorafenib arm [hazard ratio (HR) 0.66, 95% confidence interval (CI) 0.52–0.85, *p* < 0.001] [5,6,7,8].

The phase III HIMALAYA trial compared single-dose tremelimumab with regular interval durvalumab (D + T) to sorafenib in the first-line setting for advanced HCC and demonstrated an improvement in mOS (16.4 vs. 13.8 months, *p* = 0.003) [9,10]. However, there was no significant difference in the median progression-free survival (mPFS) between the D + T and sorafenib (5.4 vs. 5.6 months), although the overall response rate (ORR) was 20% in the D + T arm compared to 5% in the sorafenib arm. Based on HIMALAYA trial results, the U.S. FDA granted approval for D + T for the treatment of advanced HCC in October 2022. Recently, an updated analysis reported a 5-year OS rate of 19.6% in patients who received D + T versus 9.4% in those who received sorafenib [6,10,11].

Atezolizumab is a monoclonal antibody that targets programmed death-ligand 1 (PD-L1), thus preventing the interaction between PD-L1 expressed on tumor or immune cells and PD-1 receptors on T-cells, which restores antitumor T-cell activity. Bevacizumab is a monoclonal antibody targeting the vascular endothelial growth factor (VEGF), thereby inhibiting angiogenesis and altering the tumor microenvironment. The combination of these two therapies provides both direct immune activation and modulation of the tumor vasculature [5,7,8]. In contrast, durvalumab inhibits PD-L1-mediated immune suppression, and tremelimumab is a T-lymphocyte-associated protein 4 (CTLA-4) inhibitor that enhances early T-cell priming and expansion. Both agents are checkpoint blockade agents that provide complementary immune activation, which may enhance antitumor immune responses but can also increase the risk of immune-mediated toxicities [7]. The distinct mechanisms of each treatment agent and their synergistic effect when used in combination may influence treatment selection in clinical practice. VEGF inhibition in A + B requires consideration of bleeding risk and portal hypertension, while CTLA-4 inhibition as part of D + T may increase the risk of immune-mediated adverse events. Understanding the mechanism and the possible toxicity profiles is important when evaluating the comparative effectiveness of each combination therapy.

Currently, no prospective randomized trial is planned to compare the efficacy of A + B and D + T in patients with advanced HCC. Additionally, there are limited data comparing the patient safety events associated with either treatment regimen. Therefore, the decision to choose one of these regimens over the other is nuanced and based on cross-trial comparisons that do not fully account for differences in baseline patient and tumor characteristics, especially considering key differences in the inclusion and exclusion criteria between these two trials. Furthermore, there were differences in adverse events between the two regimens, which may have influenced the decision to choose one regimen over another. In the present study, we conducted a propensity-score-matched analysis to compare the outcomes of patients with advanced HCC receiving A + B with those receiving D + T as the initial systemic therapy in real-world settings.

## 2. Methods

### 2.1. Patient Selection

The primary aim of this study was to assess the difference in OS in patients who were categorized as BCLC-B, were ineligible to receive locoregional therapy or BCLC-C, or received A + B or D + T in the first-line setting. For this study, we used the TriNetX platform, a global federated health research network that provides access to electronic health record data from more than 112 healthcare organizations throughout the United States (TriNetX Research Network, Cambridge, MA, USA; date of data access: 7 May 2026). We identified patients aged ≥18 years with hepatocellular cancer (ICD-10: C22) and stratified them into two cohorts based on the treatment received in the first-line setting: A + B versus D + T. Patients were excluded if they had prior treatment with sorafenib (RX: 495881) or had active or untreated hepatitis virus (ICD-10: B15.9, B17.1) prior to starting A + B or D + T therapy. Patients were also excluded if they had esophageal varices with bleeding (ICD-10: I85.01) or gastrointestinal hemorrhage (ICD-10: K92.2, I85.01) 6 months prior to starting A + B or D + T therapy. Patients were required to have no inpatient encounters in the 7 days preceding therapy initiation, at least one follow-up visit after initiation, and survival of at least 30 days following therapy initiation. The index date for both cohorts was the date of therapy initiation. Demographic data, laboratory markers including serum albumin, bilirubin, and INR to serve as surrogates of liver function, and comorbidities were summarized for this study.

### 2.2. Propensity Score Matching

All analyses were conducted using the TriNetX online analytics platform. The propensity scores for receiving A + B or D + T were estimated based on the following covariates: patient demographics (age at index, sex, race, ethnicity), liver disease risk factors (history of chronic viral hepatitis ICD-10: B15-B19; or alcohol-related disorder, ICD-10: F10), obesity (BMI ≥ 30 kg/m^2^), serum albumin thresholds of <2.8 g/DL, 2.8–3.5 g/DL, and ≥3.5 g/DL, serum bilirubin thresholds of <2 mg/DL, 2–3 mg/DL, and ≥3 mg/DL, INR thresholds of <1.7, 1.7–2.3, and ≥2.3, history of ascites (ICD: R18), esophageal varices (ICD-10: I85), presence of metastatic disease (ICD-10: C77, C78.0), malignant neoplasm of extrahepatic bile duct (ICD-10: C24.0) and portal vein thrombosis (ICD-10: I81). For laboratory-based covariates, values were categorized into clinically relevant threshold groups as specified above. Patients without laboratory measurements were considered as not meeting the criteria for any category, and those with values spanning multiple categories were included in each applicable group. In TriNetX, cell counts ≤10 are reported as 10 to protect patient privacy; therefore, category totals may exceed 100% (for example, race).

A 1:1 matching was then performed using the nearest-neighbor greedy method, with a caliper of 0.1 pooled standard deviations. Covariate balance within the matched subset was evaluated using standardized mean differences (SMDs), with an SMD < 0.1 considered indicative of adequate balance between cohorts. For subgroup analyses, propensity score matching was performed separately within each subgroup to generate independent matched cohorts for each comparison.

### 2.3. Survival Analyses

Overall 6-month, 12-month and 3-year survival in the matched cohort was estimated using the Kaplan–Meier method, with between-group differences assessed using the log-rank test. The OS was calculated from the beginning of treatment with A + B or D + T until death. Patients who survived were censored on the date of their last follow-up visit. Hazard ratios (HRs) were calculated using the Cox proportional model. The statistical *p*-value was set at *p* < 0.05. Stratified subgroup analyses comparing survival A + B and D + T were conducted by race (White and non-White), presence of esophageal varices, and viral hepatitis.

### 2.4. Patient Safety Analyses

New incidences and associations were assessed for colitis, immune-mediated hepatitis (ICD-10: K75.4; K71), hypertension (ICD-10: I10) or proteinuria (ICD-10: R80), and endocrinopathies, including hypothyroidism (ICD-10: E03.9), hypopituitarism (ICD-10: E23), adrenal insufficiency (ICD-10: E27.4) or type 1 diabetes mellitus (ICD-10: E10), bleeding risk (ICD-10: K92.2 or I85.01), and hospitalization (visit: inpatient encounter or CPT: 99,221–99,223; 99,231–99,239) at 90 days and 6 months after the initiation of treatment. Results are reported as odds ratio and 95% confidence intervals. Stratified analyses of hospitalizations were conducted by race (White and non-White), presence of esophageal varices and viral hepatitis etiology.

## 3. Results

### 3.1. Baseline Characteristics

A total of 2819 patients were assessed, including 2031 who received A + B and 788 who received D + T in the first-line setting (Table 1). Compared to patients in the A + B cohort, those who received D + T were more likely to be older (68.4 vs. 66.9 years, SMD = 0.15) and have lower platelet counts (186.8 vs. 201.3, SMD = 0.13). For baseline liver function, the D + T cohort exhibited more hypoalbuminemia with levels < 2.7 g/dL (35.2% vs. 28.4%, SMD = 0.15), as well as a higher prevalence of bilirubin levels between 2.0 and 2.9 mg/dL (28.8% vs. 23.8%, SMD = 0.11), and decreased INR with levels between 0.0 and 1.6 (92.1% vs. 86.3%, SMD = 0.19) (Table 1). Those who received A + B more often had a history of viral hepatitis (47% vs. 39.2%, SMD = 0.16), whereas those who received D + T more often had a history of ascites (31.2% vs. 18.3%, SMD = 0.30), esophageal varices (29.4% vs. 21.5%, SMD = 0.18), alcohol use disorder (21.3% vs. 16.8%, SMD = 0.11), and metastatic disease (1.3% vs. 0.5%, SMD = 0.08), with metastasis to the lung being most common in the A + B cohort (5.8% vs. 3.8%, SMD = 0.09) (Table 1). Baseline characteristics within subgroup analyses demonstrated variable balance following propensity score matching (Tables S1–S4).

### 3.2. Propensity Score Matching

In the A + B cohort, 768 patients were matched with 768 patients in the D + T group. All covariates were adequately balanced in the matched set, with an SMD < 0.1 for all covariates included in PSM model (Table 1). The SMD for INR after matching in the full cohort analysis was 0.10, indicating borderline imbalance between cohorts. Before and after matching, the A + B group demonstrated longer mean follow-up compared to D + T for all comparisons (Table 1 and Tables S1–S4).

### 3.3. Survival of Matched Groups

**Figure 1:**
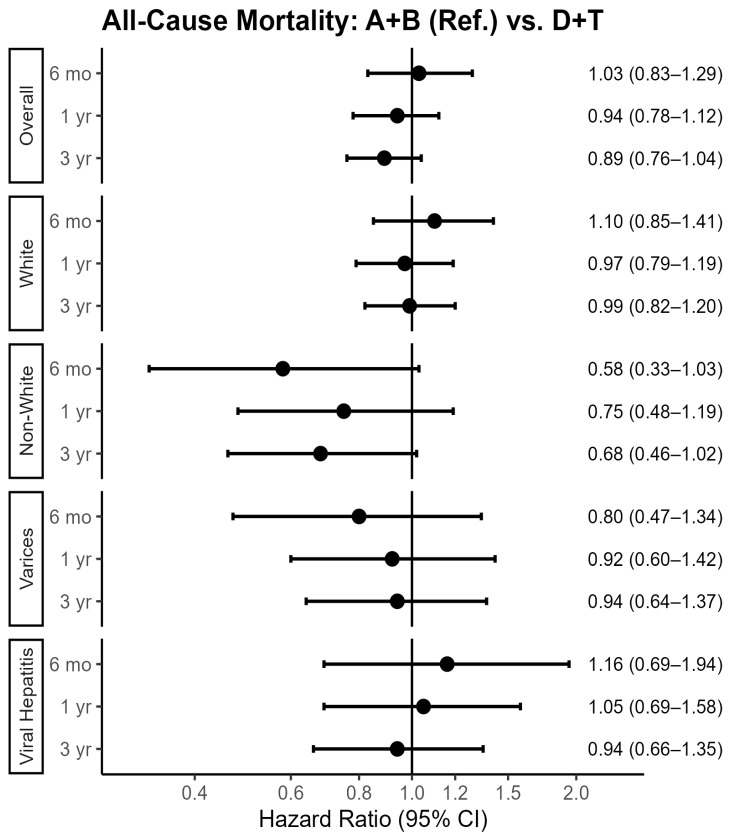
Forest plot of hazard ratios (HRs) and 95% confidence intervals (CIs) for all-cause mortality at 6 months, 1 year, and 3 years following treatment initiation. Analyses were performed within separately propensity-score-matched cohorts for the overall population and each subgroup, including White, non-White, presence of esophageal varices (i.e., varices), and viral hepatitis status (Tables S1–S4).

At six months, survival was similar between treatment groups, with estimated survival probabilities of 76.1% for A + B and 75.9% for D + T (hazard ratio (HR) 1.03; 95% confidence interval (CI), 0.83–1.29). Survival declined by 1 year, with corresponding survival estimates of 58.3% for A + B and 62.2% for D + T (HR 0.94; 95% CI, 0.78–1.12). At 3 years, there was no statistically significant difference in median overall survival between patients receiving A + B and those receiving D + T (A + B: 496 vs. D + T: 683 days; HR 0.89; 95% CI, 0.76–1.04; Figure 1).

### 3.4. Stratified Analyses by Subgroups

Race: Among White patients with HCC, survival was comparable between A + B and D + T at 6 months (A + B: 71.9% vs. D + T: 70.3%; HR 1.10; 95% CI, 0.85–1.41) and at 1 year (A + B: 52.5% vs. D + T: 55.6%, HR 0.97; 95% CI, 0.79–1.19). At 3 years, survival remained similar, with median overall survival of 403 days for A + B and 488 days for D + T (A + B: 30.3% vs. D + T: 26.8% survival probability; HR 0.99; 95% CI, 0.82–1.20; *p* = 0.93; Figure 1).

Among non-White patients, survival consistently favored D + T across time points, including at 6 months (D + T: 87.2% vs. A + B: 79.2%; HR 0.58; 95% CI, 0.33–1.03), 1 year (D + T: 74.2% vs. A + B: 65.2%; HR 0.75; 95% CI, 0.48–1.19), and 3 years (D + T: 58.3% vs. A + B: 32.8%; HR 0.68; 95% CI, 0.46–1.02; Figure 1), although these differences did not reach statistical significance.

Esophageal Varices: Among patients with esophageal varices, survival was similar between treatment groups at 6 months (A + B 65.1% vs. D + T 73.0%; HR 0.80; 95% CI, 0.47–1.34; *p* = 0.40) and at 1 year (A + B: 51.0% vs. D + T: 53.8%; HR 0.92; 95% CI, 0.60–1.42; *p* = 0.70). At 3 years, median OS was 366 days for A + B and 440 days for D + T (A + B: 21.3% vs. D + T: 18.9% survival probability; HR 0.94; 95% CI, 0.64–1.37; *p* = 0.73; Figure 1).

Viral Hepatitis: Among patients with viral hepatitis, survival outcomes were comparable between treatment groups at 6 months (A + B: 81.7% vs. D + T: 79.3%; HR 1.16; 95% CI, 0.69–1.94; *p* = 0.57) and at 1 year (A + B: 65.2% vs. D + T: 65.8%; HR 1.05; 95% CI, 0.69–1.58; *p* = 0.82). At 3 years, median OS was 555 days for A + B and 773 days for D + T (A + B: 34.5% vs. D + T: 32.1% survival probability; HR 0.94; 95% CI, 0.66–1.35; *p* = 0.74; Figure 1).

### 3.5. Second-Line Treatment Rates

A higher proportion of patients in the A + B cohort transitioned to second-line therapy with either Lenvatinib or Cabozantinib compared to the D + T cohort (19.6% vs. 14.5%). In both groups, Lenvatinib use was more prevalent, utilized by 13.6% of the A + B cohort and 11.4% of the D + T cohort. Cabozantinib was used less frequently, with rates of 2.9% and 1.8%, respectively.

### 3.6. Patient Safety of Matched Groups

Ninety days after starting therapy, in the propensity-matched cohort, D + T was associated with a higher incidence of acute immune-mediated colitis in all patients (5.1% vs. 3.0%, odds ratio (OR) 1.7, 95% CI 0.99–2.92, *p* < 0.05) and in White patients (6.2% vs. 3.4%, OR 1.88, 95% CI 0.99–3.58, *p* < 0.05). All-cause hospitalizations were high across both cohorts but did not differ significantly between A + B (30.3%) and D + T (33.3%, OR 1.15, 95% CI 0.93–1.42, *p* = 0.21). Subgroup analyses showed that White patients receiving D + T had higher rates of hospitalization as compared to those who received A + B (35% vs. 29.1%, OR 1.23, 95% CI 0.99–1.73, *p* = 0.05), whereas no disparities were noted among non-White patients (30% vs. 33.3%, OR 0.85, 95% CI 0.54–1.34, *p* = 0.49) or those with esophageal varices (37.9% vs. 39.8%, OR 0.92, 95% CI 0.53–1.61, *p* = 0.77) or viral hepatitis (27.3% vs. 19.9%, OR 1.52, 95% CI 0.90–2.55, *p* = 0.12). Rates of immune-mediated hepatitis (1.6% vs. 1.9%, OR 0.86, 95% CI 0.39–1.86, *p* = 0.69), endocrinopathies (8.3% vs. 7.8%, OR 1.06, 95% CI 0.72–1.58, *p* = 0.53) hypertension or proteinuria (13.1% vs. 18.3%, OR 0.67, 95% CI 0.41–1.09, *p* = 0.11), and bleeding risk (5.9% vs. 4.3%, OR 1.39, 95% CI 0.87–2.19, *p* = 0.16) were statistically comparable between the two cohorts at ninety days (Table 2). Within six months of starting treatment, there was no significant difference in the risk of immune-mediated hepatitis (2.5% vs. 3.1%, OR 0.82, 95% CI 0.44–1.52, *p* = 0.53), endocrinopathies (8.3% vs. 7.8%, OR 1.06, 95% CI 0.72–1.58, *p* = 0.53), and bleeding (7.3% vs. 6.8%, OR 1.08, 95% CI 0.73–1.60, *p* = 0.69) between the regimens. In the subgroup analysis of patients with esophageal varices, there was no significant difference in the bleeding risk between the cohorts (13.9% vs. 10.9%, OR 1.32, 95% CI 0.57–3.06, *p* = 0.52; Table 2).

## 4. Discussion

The treatment landscape for advanced HCC has dramatically shifted in recent years with the introduction of immunotherapy into the treatment paradigm; however, ambiguity remains as to which regimen may portend the best outcomes. In this propensity-matched analysis, we showed comparable survival among those who received A + B and those who received D + T when controlling for baseline patient and tumor factors. D + T was associated with a higher risk of acute immune-mediated colitis and hospitalization. At this time, the decision to choose one treatment over another may be more likely based on adverse effects, perceived patient risk, patient and physician preferences, and baseline clinical factors.

Recent multicenter real-world studies have similarly demonstrated comparable effectiveness between A + B and D + T. Hiraoka et al. retrospectively evaluated these regimens across multiple institutions in Japan and found no significant differences in overall survival after adjustment for baseline characteristics and highlighted that treatment selection was strongly influenced by underlying liver function and the presence of portal hypertension [12]. Similarly, Ohama et al. conducted a multicenter retrospective matched study comparing patients treated with A + B or D + T, and no significant differences were observed in overall survival between the two regimens; however, D + T showed significantly higher immune-related adverse event incidence, whereas A + B resulted in a progressive decrease in ALBI scores, suggesting differing toxicity profiles that may influence treatment selection [8]. These findings, along with our analysis, highlight the importance of real-world studies as they provide insight into treatment outcomes among broader patient populations while accounting for treatment-selection biases that are inherent in retrospective studies.

Previous studies have attempted to compare outcomes between A + B and D + T using network meta-analyses, finding similar survival rates between regimens [13,14]. However, it is challenging to make cross-trial comparisons given the important differences in the inclusion criteria between HIMALAYA and IMBrave150. For example, higher-risk patients with main portal vein tumor thrombosis were excluded from the HIMALAYA trial but were allowed to enroll in IMBrave150 [15,16]. Further, a significantly higher proportion of patients receiving D + T had a history of ascites and varices, suggesting poorer baseline hepatic function and greater portal hypertension within this cohort. This likely reflects treatment-selection bias rather than differences attributable to the therapy regimens themselves. Given the risk of VEGF inhibition-associated risk of hemorrhagic complications, bevacizumab-containing regimens may be preferentially avoided in patients with significant portal hypertension or uncontrolled varices [15]. Therefore, patients with more advanced liver disease may be more likely to receive D + T, thus contributing to differences in baseline characteristics between the treatment groups. Similarly, differences in laboratory markers reflecting hepatic function and disease burden, including albumin, bilirubin, platelet count, and AFP, may influence both treatment selection and prognosis in advanced HCC. Propensity score matching provides an important mechanism to reduce measured confounding variables and allows for a more balanced comparison between treatment groups, as differences in baseline hepatic function are known to significantly influence survival outcomes in patients with HCC [15].

Further impacting treatment decisions in advanced HCC is the consideration of recent updates from the HIMALAYA trial, which reported an improvement in 5-year OS with D + T compared to sorafenib [16]. This is the first trial in HCC reporting 5-year OS data, although the finding of long-term survival benefit with doublet immunotherapy is consistent with that observed in other malignancies, including melanoma and renal cell carcinoma [17,18].

Our study also included subgroup analyses. In an analysis among White vs. non- White patients, we found that survival outcomes were generally comparable among White patients between the cohorts. For non-White patients, several survival estimates were unable to be reported, likely due to the limited sample size or relatively low events seen within TriNetX. Therefore, we are unable to say if there are potential differences in treatment response across racial groups. While these results suggest a lack of significant differences in our study, Lau-Min et al. found that Asian and Hispanic patients achieved better OS on systemic therapies compared to White patients, with mOS rates of 10.3 and 10.5 months as compared to 8.0 months, respectively [19]. This discrepancy highlights the importance of future studies to determine if survival advantages are in part due to either biologic factors or treatment accessibility.

Furthermore, when deciding between regimens, one must consider the unique toxicity profiles of each. In the case of A + B, bevacizumab, a VEGF inhibitor, increased the risk of bleeding [15,17,18]. As a result, the IMBrave150 trial required patients to undergo upper endoscopy prior to treatment and excluded patients with untreated or incompletely treated esophageal varices [20,21,22]. In an additional subgroup analysis that we conducted, among patients with esophageal varices or underlying viral hepatitis, survival outcomes remained comparable between the treatment regimens, with no statistical difference observed at 6 months, 1 year, or 3 years. With these subgroup analyses, the findings further suggest that there is comparable effectiveness in terms of survival, while toxicity profiles remain an important consideration when selecting between A + B and D + T. While the presence of varices historically has created concern with the use of VEGF inhibitors like bevacizumab, our data aligns with recent findings from Park et al., suggesting that with proactive measures including upper endoscopy and variceal management, patients can safely undergo treatment with comparable survival outcomes and no increase in bleeding risk [23].

One limitation of this study is the inability to account for all factors including Child–Pugh score, ascites grade (although we were able to determine whether a patient had a history of ascites) and encephalopathy within the TriNetX platform. However, the ALBI score, which utilizes only two objective variables, albumin and bilirubin, to assess liver function, has shown prognostic value in patients with HCC and may even distinguish between two different prognostic subpopulations among those with Child–Pugh class A liver function [14,16].

Furthermore, we did not have data available to calculate the response rates or PFS. We do know that patients who respond to treatment tend to have longer survival times; however, in HCC, an increase in ORR and PFS does not necessarily translate to survival improvement. In the HIMALAYA trial, the D + T regimen demonstrated an improvement in OS compared to sorafenib, although there was no difference in mPFS between the two groups [11]. Especially in this retrospective study, OS alone may be influenced by subsequent treatment after first-line therapy. TriNetX does not support reliable time-to-outcome events such as time of treatment or time to treatment discontinuation. Therefore, as a supplementary measure, we reported the proportion of patients who received subsequent second-line therapy with either Lenvatinib or Cabozantinib after receiving either A + B or D + T initially. We found that a higher proportion of patients on A + B transitioned to Lenvatinib or Cabozantinib as compared to those taking D + T. This could potentially suggest earlier discontinuation or progression as compared to D + T [24]. Lau Min et al. emphasize in their study that only approximately 20.7% of patients received second-line therapy, with mOS being 8.1 months, but this was not in correlation with the type of first-line treatment choice, and, therefore, the challenge of optimal sequencing of treatment agents to maximize OS and PFS still remains [25].

Another limitation in this observational study is the ability to include relevant factors such as liver function, tumor burden, and vascular invasion in the analyses. To account for residual confounding factors, we incorporated propensity score matching for clinically meaningful laboratory thresholds for baseline liver function, including INR, albumin, and bilirubin. We have also propensity-score-matched for metastatic disease to the lungs and lymph nodes for each cohort and reflected advanced disease by incorporating portal vein thrombosis. With all variables adequately matched for the final analyses, we were able to partially mitigate imbalances for baseline covariates. However, we acknowledge that unmeasured factors such as performance status and detailed tumor burden may still contribute to the study findings. Despite the fact that albumin and bilirubin were accounted for in this study, we were unable to calculate ALBI score due to limitation of the TriNetX platform. Other limitations of our study include its retrospective nature and the inclusion of patients from only healthcare organizations that enrolled on the TriNetX platform, which may limit the generalizability of its conclusions. However, the included patients were from different regions of the world, which broadens the applicability of the study results. This is especially true for Western and Asian populations, as the etiology of cirrhosis and outcomes differ in these two groups.

## 5. Conclusions

In conclusion, in this propensity-matched analysis accounting for patient characteristics and markers of liver function, we showed comparable survival among patients with advanced HCC who received A + B and D + T. This suggests that both may be valid first-line therapeutic options for advanced HCC and that the choice of therapy is more likely to depend on patient and tumor characteristics, drug toxicities, and physician and patient preferences. To our knowledge, this is the largest study comparing the outcomes of these regimens in a real-world setting, and while a randomized controlled trial is ideal, no prospective clinical trial is being planned at this time.

## Figures and Tables

**Table 1:** Baseline characteristics of HCC patients treated with atezolizumab plus bevacizumab (A + B) or durvalumab plus tremelimumab (D + T) for 90-day, 6-month, 1-year, and 3-year analyses, before and after propensity score matching.

	Before Matching			After Matching		
Baseline Characteristic	A + B N = 2031 ^1,2^	D + T N = 788 ^1,2^	SMD ^3^	A + B N = 768 ^1,2^	D + T N = 768 ^1,2^	SMD ^3^
***Age at index***	66.99 ± 9.40	68.39 ± 9.38	**0.15**	68.17 ± 8.47	68.34 ± 9.37	0.02
***Sex***						
Female	429 (21.12%)	152 (19.29%)	0.05	137 (17.84%)	151 (19.66%)	0.05
Male	1601 (78.83%)	635 (80.58%)	0.04	630 (82.03%)	616 (80.21%)	0.05
***Race***						
American Indian or Alaska Native	13 (0.64%)	11 (1.4%)	0.08	≤10 (1.3%)	≤10 (1.3%)	<0.01
Asian	290 (14.28%)	82 (10.41%)	**0.12**	78 (10.16%)	82 (10.68%)	0.02
Black or African American	243 (11.97%)	88 (11.17%)	0.02	89 (11.59%)	88 (11.46%)	<0.01
Native Hawaiian or Other Pacific Islander	12 (0.59%)	≤10 (1.27%)	0.07	≤10 (1.3%)	≤10 (1.3%)	<0.01
Other race	90 (4.43%)	57 (7.23%)	**0.12**	51 (6.64%)	49 (6.38%)	0.01
Unknown race	262 (12.9%)	62 (7.87%)	**0.17**	60 (7.81%)	62 (8.07%)	0.01
White	1121 (55.19%)	486 (61.68%)	**0.13**	476 (61.98%)	475 (61.85%)	<0.01
***Ethnicity***						
Hispanic or Latino	141 (6.94%)	84 (10.66%)	**0.13**	80 (10.42%)	77 (10.03%)	0.01
Not Hispanic or Latino	1292 (63.61%)	512 (64.98%)	0.03	487 (63.41%)	502 (65.37%)	0.04
Unknown ethnicity	598 (29.44%)	192 (24.37%)	**0.11**	201 (26.17%)	189 (24.61%)	0.04
***Labs***						
Platelets [number/volume] in blood ^4^	201.3 ± 111.6	186.7 ± 116.0	**0.13**	191.8 ± 106.3	187.7 ± 115.0	0.04
Platelet lab n	1890	753		733	733	
Albumin [mass/volume] in serum, Plasma or blood	3.6 ± 0.6	3.5 ± 0.6	**0.20**	3.5 ± 0.6	3.5 ± 0.6	0.06
Albumin lab n	1817	740		709	720	
Albumin 0–2.7 g/dL	576 (28.36%)	277 (35.15%)	**0.15**	241 (31.38%)	264 (34.38%)	0.06
Albumin 2.8–3.4 g/dL	1279 (62.97%)	559 (70.94%)	**0.17**	539 (70.18%)	541 (70.44%)	0.01
Albumin ≥ 3.5 g/dL	1640 (80.75%)	660 (83.76%)	0.08	624 (81.25%)	643 (83.72%)	0.07
Bilirubin total [mass/volume] in serum, plasma or blood	1.2 ± 1.2	1.2 ± 1.2	0.06	1.3 ± 1.3	1.2 ± 1.2	0.02
Bilirubin lab n	1836	734		707	717	
Bilirubin 0–1.9 mg/dL	1788 (88.04%)	707 (89.72%)	0.05	680 (88.54%)	692 (90.1%)	0.05
Bilirubin 2–2.9 mg/dL	484 (23.83%)	227 (28.81%)	**0.11**	211 (27.47%)	218 (28.39%)	0.02
Bilirubin ≥ 3 mg/dL	277 (13.64%)	125 (15.86%)	0.06	112 (14.58%)	120 (15.63%)	0.03
INR in plasma or blood	1.2 ± 0.3	1.2 ± 0.3	**0.12**	1.2 ± 0.2	1.2 ± 0.3	0.07
INR lab n	1756	727		705	707	
INR 0–1.6	1753 (86.31%)	726 (92.13%)	**0.19**	704 (91.67%)	706 (91.93%)	0.01
INR 1.7–2.2	155 (7.63%)	95 (12.06%)	**0.15**	85 (11.07%)	88 (11.46%)	0.01
INR ≥ 2.3	83 (4.09%)	32 (4.06%)	0.00	31 (4.04%)	32 (4.17%)	0.01
BMI kg/m^2^	27.5 ± 5.8	27.9 ± 5.9	0.07	27.7 ± 5.4	27.9 ± 5.9	0.03
BMI, n	1730	695		663	675	
BMI ≥ 30 kg/m^2^, n (%)	841 (41.41%)	363 (46.07%)	**0.09**	346 (45.05%)	351 (45.7%)	0.01
Alpha-1-Fetoprotein ^4^	4346.5 ± 12,799.0	3077.2 ± 9179.0	**0.11**	4156.2 ± 12,613.1	3011.3 ± 9063.6	**0.10**
Alpha-1-Fetoprotein lab n	904	372		368	362	
**Comorbidities**						
Viral hepatitis (B15–B19)	955 (47.02%)	309 (39.21%)	**0.16**	308 (40.1%)	307 (39.97%)	<0.01
Ascites (R18)	372 (18.32%)	246 (31.22%)	**0.30**	224 (29.17%)	230 (29.95%)	0.02
Esophageal varices (I85)	437 (21.52%)	232 (29.44%)	**0.18**	213 (27.73%)	218 (28.39%)	0.01
Alcohol-related disorders (F10)	342 (16.84%)	168 (21.32%)	**0.11**	167 (21.75%)	160 (20.83%)	0.02
Portal vein thrombosis (I81)	329 (16.2%)	147 (18.66%)	0.06	141 (18.36%)	145 (18.88%)	0.01
Malignant neoplasm of extrahepatic bile duct (C24.0)	≤10 (0.49%)	≤10 (1.27%)	**0.08**	≤10 (1.3%)	≤10 (1.3%)	<0.01
**Secondary Malignancy**						
Secondary and unspecified malignant neoplasm of lymph nodes (C77)	136 (6.7%)	41 (5.2%)	0.06	41 (5.34%)	40 (5.21%)	0.01
Secondary malignant neoplasm of lung (C78.0)	117 (5.76%)	30 (3.81%)	**0.09**	25 (3.26%)	30 (3.91%)	0.04
**Mean Follow-up Time (Days)**						
**90-day analysis**	85.20 ± 14.96	80.56 ± 20.59	NA	86.08 ± 13.29	80.55 ±20.58	NA
**6-month analysis**	156.07 ± 45.11	141.25 ± 55.79	NA	157.99 ± 42.54	141.22 ± 55.8	NA
**1-year analysis**	263.14 ± 119.38	219.92 ± 126.59	NA	264.79 ± 115.88	219.81 ± 126.60	NA
**3-year analysis**	444.53 ± 343.85	295.71 ± 250.86	NA	436.47 ± 333.06	296.4 ± 252.09	NA

^1^ Mean ± SD; n (%); ^2^ cell counts ≤10 are reported as ≤10 in accordance with TriNetX privacy protection policies; totals may not equal 100% and may exceed 100% due to small cell counts and category omission; ^3^ standardized mean differences (SMDs) are presented; bold values of >0.1 indicate imbalance between cohorts; ^4^ platelets and Alpha-1-Fetoprotein not included in propensity score matching. Laboratory values were categorized using predefined thresholds. Patients without laboratory measurements were considered as not meeting any category, and those with values across multiple ranges were included in each applicable category. Consequently, proportions may exceed 100%.

**Table 2:** Adverse events experienced by HCC patients, stratified by treatment with A + B vs. D + T after propensity score matching.

Adverse Event	Cohort	Patients in Cohort ^1,2^	Patients with Outcome ^1,2^	Risk %	OR (95% CI)	*p*-Value
***Colitis*** ^3^						
*All Patients*	A + B	724	22	3.0%		
	D + T	729	37	5.1%	1.71 (0.99, 2.92)	0.049
*Non-White Patients * ^5^	A + B	NA	NA	NA		
	D + T	NA	NA	NA	NA	NA
*White Patients*	A + B	440	15	3.4%		
	D + T	449	28	6.2%	1.88 (0.99, 3.58)	0.049
*Esophageal Varice Patients*	A + B	NA	NA	NA		
	D + T	NA	NA	NA	NA	NA
*Viral Hepatitis Patients*	A + B	NA	NA	NA		
	D + T	NA	NA	NA	NA	NA
***Hospitalization *** **^1,3^**						
*All Patients*	A + B	768	233	30.3%		
	D + T	768	256	33.3%	1.15 (0.93, 1.42)	0.21
*Non-White Patients * ^5^	A + B	174	58	33.3%		
	D + T	174	52	29.9%	0.85 (0.54, 1.34)	0.49
*White Patients*	A + B	471	137	29.1%		
	D + T	471	165	35.0%	1.32 (0.99, 1.73)	0.051
*Esophageal Varice Patients*	A + B	103	41	39.8%		
	D + T	103	39	37.9%	0.92 (0.53, 1.61)	0.77
*Viral Hepatitis Patients*	A + B	161	32	19.9%		
	D + T	161	44	27.3%	1.52 (0.90, 2.55)	0.12
***Hypertension/proteinuria *** **^4^**						
*All Patients*	A + B	257	47	18.3%		
	D + T	245	32	13.1%	0.67 (0.41, 1.09)	0.11
*Non-White Patients * ^5^	A + B	NA	NA	NA		
	D + T	NA	NA	NA	NA	NA
*White Patients*	A + B	136	27	19.9%		
	D + T	125	18	14.4%	0.68 (0.35, 1.31)	0.24
*Esophageal Varice Patients*	A + B	NA	NA			
	D + T	NA	NA	NA	NA	NA
*Viral Hepatitis Patients*	A + B	NA	NA			
	D + T	NA	NA	NA	NA	NA
***Immune-Mediated Hepatitis *** **^3^**						
*All Patients*	A + B	755	14	1.9%	0.86 (0.39, 1.86)	0.69
	D + T	754	12	1.6%		
***Immune-Mediated Hepatitis *** **^4^**						
*All Patients*	A + B	755	23	3.1%	0.82 (0.44, 1.52)	0.53
	D + T	754	19	2.5%		
***Endocrinopathies *** **^3,6^**						
*All Patients*	A + B	665	26	3.91%	1.00 (0.57, 1.74))	>0.99
	D + T	665	26	3.91%		
***Endocrinopathies *** **^4,6^**						
*All Patients*	A + B	665	52	7.8%		
	D + T	665	55	8.3%	1.06 (0.72, 1.58)	0.76
*Non-White Patients * ^5^	NA	NA	NA			
	NA	NA	NA	NA	NA	NA
*White Patients*	A + B	395	28	7.1%		
	D + T	396	34	8.6%	1.23 (0.73, 2.07)	0.43
*Esophageal Varice Patients*	A + B	NA	NA			
	D + T	NA	NA	NA	NA	NA
*Viral Hepatitis Patients*	A + B	NA	NA			
	D + T	NA	NA	NA	NA	NA
***Bleeding Risk *** **^1,3^**						
*All Patients*	A + B	768	33	4.3%		
	D + T	768	45	5.9%	1.39 (0.87, 2.19)	0.16
*Non-White Patients * ^5^	A + B	NA	NA	NA		
	D + T	NA	NA	NA	NA	NA
*White Patients*	A + B	471	18	3.8%		
	D + T	471	28	5.9%	1.59 (0.87, 2.92)	0.13
*Esophageal Varice Patients*	A + B	NA	NA			
	D + T	NA	NA	NA	NA	NA
*Viral Hepatitis Patients*	A + B	NA	NA			
	D + T	NA	NA	NA	NA	NA
***Bleeding Risk *** **^1,4^**						
*All Patients*	A + B	768	52	6.8%		
	D + T	768	56	7.3%	1.08 (0.73, 1.60)	0.69
*Non-White Patients * ^5^	A + B	NA	NA	NA		
	D + T	NA	NA	NA	NA	NA
*White Patients*	A + B	471	28	5.9%		
	D + T	471	34	7.2%	1.23 (0.73, 2.07)	0.43
*Esophageal Varice Patients*	A + B	101	11	10.9%		
	D + T	101	14	13.9%	1.32 (0.57, 3.06)	0.52
*Viral Hepatitis Patients*	A + B	NA	NA			
	D + T	NA	NA	NA	NA	NA

^1^ Denominators vary by outcome due to exclusion of patients with prior events. For bleeding risk, only pre-existing diagnoses within the 6-month pre-treatment period were excluded. Hospitalizations occurring within 7 days prior to treatment initiation were excluded; ^2^ NA indicates results could not be reported due to low event counts (≤10) in accordance with TriNetX privacy reporting thresholds.; ^3^ denotes incidence of the event within 90 days of treatment initiation; ^4^ denotes incidence of the event within 6 months of treatment initiation; ^5^ non-White includes Asian, American Indian or Alaska Native, Black or African American, Native Hawaiian or Other Pacific Islander; ^6^ endocrinopathies include hypothyroidism, hypopituitarism, adrenal insufficiency, and type 1 diabetes mellitus.

## Data Availability

Restrictions apply to the availability of these data. Minimal, aggregate, de-identified datasets used for baseline characteristics and primary outcomes were obtained from TriNetX and are available upon request from the authors with the permission of TriNetX. The data are not publicly available due to restrictions from TriNetX platform.

## Supplementary Materials

The following supporting information can be downloaded at https://www.mdpi.com/article/10.3390/biomedicines14071638/s1, Table S1. Baseline characteristics of White HCC patients treated with Atezolizumab plus Bevacizumab (A + B) or Durvalumab plus Tremelimumab (D + T), before and after propensity score matching. Table S2. Baseline characteristics of Non-White HCC patients treated with Atezolizumab plus Bevacizumab (A + B) or Durvalumab plus Tremelimumab (D + T), before and after propensity score matching. Table S3. Baseline characteristics of Esophageal varices HCC patients treated with Atezolizumab plus Bevacizumab (A + B) or Durvalumab plus Tremelimumab (D + T), before and after propensity score matching. Table S4. Baseline characteristics of Viral Hepatitis HCC patients treated with Atezolizumab plus Bevacizumab (A + B) or Durvalumab plus Tremelimumab (D + T), before and after propensity score matching.
