# Supplementary material for: Atezolizumab Plus Bevacizumab Versus Durvalumab Plus Tremelimumab for Advanced Hepatocellular Carcinoma: A Propensity-Score-Matched Analysis
Source: Biomedicines. 2026 Jul 21;14(7):1638. doi: 10.3390/biomedicines14071638 (PMC13406657; doi:10.3390/biomedicines14071638)
Supplement: Supplementary file 1 [file biomedicines-14-01638-s001.zip › biomedicines-4423265-supplementary.pdf]

Table S1. Baseline characteristics of White HCC patients treated with Atezolizumab plus Bevacizumab (A + B) or Durvalumab plus Tremelimumab (D + T), before and after propensity score matching.

	Before Matching			After Matching		
**Baseline characteristic**	**White: A+B** N =1,121[1,2]	**White: D+T** N =486[1,2]	**SMD[3]**	**White: A+B** N = 471[1,2]	**White: D+T** N =471[1,2]	**SMD[3]**
Age at index	67.93 ± 8.45	69.04 ± 9.07	**0.13**	68.49 ± 8.41	68.97 ± 9.11	0.05
Sex						
Female	223 (19.89%)	94 (19.34%)	0.01	90 (19.11%)	92 (19.53%)	0.01
Male	897 (80.02%)	391 (80.45%)	0.01	381 (80.89%)	378 (80.26%)	0.02
Race						
American Indian or Alaska Native	0 (0%)	0 (0%)		0 (0%)	0 (0%)	
Asian	0 (0%)	0 (0%)		0 (0%)	0 (0%)	
Black or African American	0 (0%)	0 (0%)		0 (0%)	0 (0%)	
Native Hawaiian or Other Pacific Islander	0 (0%)	0 (0%)		0 (0%)	0 (0%)	
Other Race	0 (0%)	0 (0%)		0 (0%)	0 (0%)	
Unknown Race	0 (0%)	0 (0%)		0 (0%)	0 (0%)	
White	1121 (100%)	486 (100%)		471 (100%)	471 (100%)	
Ethnicity						
Hispanic or Latino	87 (7.76%)	50 (10.29%)	0.09	46 (9.77%)	50 (10.62%)	0.03
Not Hispanic or Latino	787 (70.21%)	349 (71.81%)	0.04	343 (72.82%)	336 (71.34%)	0.03
Unknown Ethnicity	247 (22.03%)	87 (17.9%)	**0.10**	82 (17.41%)	85 (18.05%)	0.02
Labs						
Platelets [#/volume] in Blood[4]	193.67 ± 110.49	171.42 ± 111.12	**0.20**	188.46 ± 103.88	172.9 ± 111.4	**0.14**
Platelet Lab n	1084	481		463	466	
Albumin [Mass/volume] in Serum, Plasma or Blood	3.59 ± 0.56	3.49 ± 0.56	**0.18**	3.56 ± 0.59	3.49 ± 0.56	**0.13**
Albumin Lab n	1063	473		454	458	
0-2.7 g/dL	344 (30.69%)	175 (36.01%)	**0.11**	168 (35.67%)	165 (35.03%)	0.01
2.8-3.4g/dL	776 (69.22%)	358 (73.66%)	**0.10**	339 (71.98%)	345 (73.25%)	0.03
≥ 3.5 g/dL	961 (85.73%)	428 (88.07%)	0.07	418 (88.75%)	414 (87.9%)	0.03
Bilirubin.total [Mass/volume] in Serum, Plasma or Blood	1.17 ± 1.17	1.33 ± 1.31	**0.12**	1.27 ± 1.3	1.31 ± 1.26	0.03
Bilirubin Lab n	1062	469		453	455	
0-1.9 mg/dL	1033 (92.15%)	448 (92.18%)	0.00	442 (93.84%)	436 (92.57%)	0.05
2-2.9 mg/dL	302 (26.94%)	150 (30.86%)	0.09	142 (30.15%)	144 (30.57%)	0.01
≥ 3 mg/dL	167 (14.9%)	79 (16.26%)	0.04	76 (16.14%)	75 (15.92%)	0.01
INR in Plasma or Blood	1.19 ± 0.29	1.21 ± 0.26	0.08	1.2 ± 0.31	1.21 ± 0.26	0.02
INR Lab n	991	451		438	436	
0-1.6 {INR}	989 (88.23%)	450 (92.59%)	**0.15**	438 (92.99%)	435 (92.36%)	0.02
1.7-2.2 {INR}	110 (9.81%)	68 (13.99%)	**0.13**	63 (13.38%)	60 (12.74%)	0.02
≥2.3 {INR}	59 (5.26%)	23 (4.73%)	0.02	27 (5.73%)	22 (4.67%)	0.05

BMI kg/m[2]	28.46 ± 5.89	28.99 ± 6.08	0.09	27.82 ± 5.57	28.99 ± 6.14	**0.20**
BMI lab n	1018	426		418	414	
At least 30 kg/m[2]	584 (52.1%)	253 (52.06%)	0.00	225 (47.77%)	248 (52.65%)	**0.10**
Alpha-1-Fetoprotein [Mass/volume] in Serum, Plasma or Blood[4]	3328.23 ± 11153.48	2579.36 ± 7635.49	0.08	3932.93 ± 11744.85	2601.59 ± 7746.54	**0.13**
Alpha-1-Fetoprotein Lab n	462	212		209	205	
**Comorbidities**						
Viral hepatitis	431 (38.45%)	164 (33.75%)	0.10	179 (38%)	162 (34.4%)	0.08
Ascites	262 (23.37%)	175 (36.01%)	0.28	177 (37.58%)	160 (33.97%)	0.08
Esophageal varices	309 (27.57%)	163 (33.54%)	0.13	158 (33.55%)	154 (32.7%)	0.02
Alcohol related disorders	248 (22.12%)	113 (23.25%)	0.03	113 (23.99%)	111 (23.57%)	0.01
Portal vein thrombosis	205 (18.29%)	103 (21.19%)	0.07	100 (21.23%)	95 (20.17%)	0.03
Malignant neoplasm of extrahepatic bile duct	10 (0.89%)	10 (2.06%)	0.10	10 (2.12%)	10 (2.12%)	0.00
**Secondary Malignancy**						
Secondary and unspecified malignant neoplasm of lymph nodes (C77)	82 (7.32%)	27 (5.56%)	0.07	31 (6.58%)	27 (5.73%)	0.04
Secondary malignant neoplasm of lung (C78.0)	57 (5.09%)	19 (3.91%)	0.06	20 (4.25%)	18 (3.82%)	0.02

[1] Mean + SD; n (%); [2] Cell counts ≤10 are reported as ≤10 in accordance with TriNetX privacy protection policies; totals may not equal 100% and may exceed 100% due to small cell counts and category omission; [3] Standardized mean differences (SMDs) are presented; bold values of > 0.1 indicate imbalance between cohorts; [4]Platelets and Alpha-1-Fetoprotein not included in propsenity score matching.

Table S2. Baseline characteristics of Non-White HCC patients treated with Atezolizumab plus Bevacizumab (A + B) or Durvalumab plus Tremelimumab (D + T), before and after propensity score matching.

	**Before Matching**			**After Matching**		
**Baseline characteristic**	**Non-White: A+B** N =558[1,2]	**Non-White D+T** N =183[1,2]	**SMD[3]**	**Non-White A+B** N = 174[1,2]	**Non-White D+T** N =174[1,2]	**SMD[3]**
Age at index	65.27 ± 9.86	67.22 ± 9.35	**0.20**	66.38 ± 8.74	67.33 ± 9.42	**0.10**
Sex						
Female	132 (23.66%)	42 (22.95%)	0.02	31 (17.82%)	42 (24.14%)	**0.16**
Male	426 (76.34%)	141 (77.05%)	0.02	143 (82.18%)	132 (75.86%)	**0.16**
Race						
American Indian or Alaska Native	13 (2.33%)	11 (6.01%)	**0.18**	≤10 (5.75%)	≤10 (5.75%)	<0.01
Asian	290 (51.97%)	82 (44.81%)	**0.14**	85 (48.85%)	78 (44.83%)	0.08
Black or African American	243 (43.55%)	88 (48.09%)	0.09	78 (44.83%)	86 (49.43%)	0.09
Native Hawaiian or Other Pacific Islander	12 (2.15%)	≤10 (5.46%)	**0.17**	≤10 (5.75%)	≤10 (5.75%)	<0.01
Other Race	0 (0%)	0 (0%)		0 (0%)	0 (0%)	
Unknown Race	0 (0%)	0 (0%)		0 (0%)	0 (0%)	
White	0 (0%)	0 (0%)		0 (0%)	0 (0%)	
Ethnicity						
Hispanic or Latino	≤10 (1.79%)	≤10 (5.46%)	**0.20**	≤10 (5.75%)	≤10 (5.75%)	<0.01
Not Hispanic or Latino	471 (84.41%)	147 (80.33%)	**0.11**	144 (82.76%)	142 (81.61%)	0.03
Unknown Ethnicity	85 (15.23%)	32 (17.49%)	0.06	29 (16.67%)	31 (17.82%)	0.03
Labs						
Platelets [#/volume] in Blood[4]	219.78 ± 114.51	218.14 ± 118.51	0.01	209.98 ± 101.18	218.74 ± 118.4	0.08
Platelet Lab n	550	183		172	174	
Albumin [Mass/volume] in Serum, Plasma or Blood	3.7 ± 0.57	3.57 ± 0.62	**0.21**	3.66 ± 0.58	3.58 ± 0.62	**0.13**
Albumin Lab n	547	179		172	170	
0-2.7 g/dL	171 (30.65%)	75 (40.98%)	**0.22**	57 (32.76%)	68 (39.08%)	**0.13**
2.8-3.4g/dL	376 (67.38%)	141 (77.05%)	**0.22**	122 (70.12%)	132 (75.86%)	**0.13**
≥ 3.5 g/dL	498 (89.25%)	160 (87.43%)	0.06	153 (87.93%)	151 (86.78%)	0.03
Bilirubin.total [Mass/volume] in Serum, Plasma or Blood	1.1 ± 1.2	1.05 ± 0.84	0.12	1.08 ± 1.09	1.05 ± 0.84	0.03
Bilirubin Lab n	518	167		157	158	
0-1.9 mg/dL	512 (91.76%)	166 (90.71%)	**0.12**	154 (88.51%)	157 (90.23%)	0.06
2-2.9 mg/dL	126 (22.58%)	48 (26.23%)	**0.13**	33 (18.97%)	43 (24.71%)	**0.14**
≥ 3 mg/dL	66 (11.83%)	26 (14.21%)	**0.10**	20 (11.49%)	25 (14.37%)	0.09
INR in Plasma or Blood	1.14 ± 0.21	1.18 ± 0.49	0.05	1.12 ± 0.14	1.18 ± 0.5	**0.17**
INR Lab n	515	174		164	165	
0-1.6 {INR}	514 (92.12%)	174 (95.08%)	**0.20**	164 (94.25%)	165 (94.83%)	0.03
1.7-2.2 {INR}	30 (5.38%)	16 (8.74%)	0.04	≤10 (5.75%)	15 (8.62%)	**0.11**
≥2.3 {INR}	19 (3.41%)	≤10 (5.46%)		≤10 (5.75%)	≤10 (5.75%)	<0.01

BMI kg/m^2	25.53 ± 5.26	25.79 ± 5.07	0.04	25.99 ± 5.21	25.82 ± 5.1	0.03
BMI lab n	481	171	0.09	154	163	
At least 30 kg/m^2	162 (29.03%)	70 (38.25%)	0.07	64 (36.78%)	67 (38.51%)	0.04
Alpha-1-Fetoprotein [Mass/volume] in Serum, Plasma or Blood[4]	6130.26 ± 15992.72	5077.18 ± 12799.64	0.07	3216.94 ± 11546.59	4596.8 ± 11222.65	**0.12**
Alpha-1-Fetoprotein Lab n	308	101		94	96	
**Comorbidities**						
Viral hepatitis	382 (68.46%)	115 (62.84%)	**0.12**	112 (64.37%)	110 (63.22%)	0.02
Ascites	81 (14.52%)	47 (25.68%)	**0.28**	42 (24.14%)	41 (23.56%)	0.01
Esophageal varices	96 (17.2%)	44 (24.04%)	**0.17**	47 (27.01%)	40 (22.99%)	0.09
Alcohol related disorders	78 (13.98%)	39 (21.31%)	**0.19**	36 (20.69%)	32 (18.39%)	0.06
Portal vein thrombosis	93 (16.67%)	32 (17.49%)	0.02	26 (14.94%)	30 (17.24%)	0.06
Malignant neoplasm of extrahepatic bile duct	≤10 (1.79%)	0 (0%)	**0.19**	0 (0%)	0 (0%)	
**Secondary Malignancy**						
Secondary and unspecified malignant neoplasm of lymph nodes (C77)	45 (8.07%)	≤10 (5.46%)	**0.10**	≤10 (5.75%)	≤10 (5.75%)	<0.01
Secondary malignant neoplasm of lung (C78.0)	38 (6.81%)	≤10 (5.46%)	0.06	≤10 (5.75%)	≤10 (5.75%)	<0.01

[1] Mean + SD; n (%); [2] Cell counts ≤10 are reported as ≤10 in accordance with TriNetX privacy protection policies; totals may not equal 100% and may exceed 100% due to small cell counts and category omission; [3] Standardized mean differences (SMDs) are presented; bold values of > 0.1 indicate imbalance between cohorts; [4]Platelets and Alpha-1-Fetoprotein not included in propsenity score matching.

Table S3. Baseline characteristics of Esophageal varices HCC patients treated with Atezolizumab plus Bevacizumab (A + B) or Durvalumab plus Tremelimumab (D + T), before and after propensity score matching.

	Before Matching			After Matching		
**Baseline characteristic**	**Varices A+B** N =197[1,2]	**Varices D+T** N =128[1,2]	**SMD[3]**	**Varices A+B** N = 103[1,2]	**Varices D+T** N =103[1,2]	**SMD[3]**
Age at index	65.54 ± 8.07	66.41 ± 8.05	**0.11**	65.56 ± 8.14	66.35 ± 8.23	**0.10**
Sex						
Female	45 (22.84%)	26 (20.31%)	0.06	24 (23.3%)	24 (23.3%)	<0.01
Male	152 (77.16%)	102 (79.69%)	0.06	79 (76.7%)	79 (76.7%)	<0.01
Race						
American Indian or Alaska Native	≤10 (5.08%)	≤10 (7.81%)	**0.11**	≤10 (9.71%)	≤10 (9.71%)	<0.01
Asian	15 (7.61%)	≤10 (7.81%)	0.01	≤10 (9.71%)	≤10 (9.71%)	<0.01
Black or African American	19 (9.65%)	≤10 (7.81%)	0.06	≤10 (9.71%)	≤10 (9.71%)	<0.01
Native Hawaiian or Other Pacific Islander	≤10 (5.08%)	0 (0%)	0.33	0 (0%)	0 (0%)	
Other Race	12 (6.09%)	≤10 (7.81%)	0.07	≤10 (9.71%)	≤10 (9.71%)	<0.01
Unknown Race	≤10 (5.08%)	≤10 (7.81%)	**0.11**	≤10 (9.71%)	≤10 (9.71%)	<0.01
White	142 (72.08%)	97 (75.78%)	0.08	76 (73.79%)	80 (77.67%)	0.09
Ethnicity						
Hispanic or Latino	27 (13.71%)	22 (17.19%)	**0.10**	22 (21.36%)	16 (15.53%)	**0.15**
Not Hispanic or Latino	139 (70.56%)	86 (67.19%)	0.07	62 (60.19%)	70 (67.96%)	**0.16**
Unknown Ethnicity	31 (15.74%)	20 (15.63%)	<0.01	19 (18.45%)	17 (16.51%)	0.05
Labs						
Platelets [#/volume] in Blood[4]	129.14 ± 75.95	118.73 ± 83.67	**0.13**	126.63 ± 76.68	121.36 ± 89.17	0.06
Platelet Lab n	194	128		101	103	
Albumin [Mass/volume] in Serum, Plasma or Blood	3.42 ± 0.52	3.28 ± 0.53	**0.26**	3.33 ± 0.53	3.33 ± 0.53	0.01
Albumin Lab n	193	124		100	99	
0-2.7 g/dL	97 (49.24%)	75 (58.59%)	**0.19**	56 (54.37%)	56 (54.37%)	<0.01
2.8-3.4g/dL	169 (85.79%)	111 (86.72%)	0.03	86 (83.5%)	88 (85.44%)	0.05
≥ 3.5 g/dL	172 (87.31%)	108 (84.38%)	0.08	86 (83.5%)	90 (87.38%)	0.11
Bilirubin.total [Mass/volume] in Serum, Plasma or Blood	1.61 ± 1.2	1.85 ± 1.63	**0.17**	1.63 ± 0.9	1.81 ± 1.71	0.13
Bilirubin Lab n	192	121		99	98	
0-1.9 mg/dL	186 (94.42%)	114 (89.06%)	**0.20**	94 (91.26%)	94 (91.26%)	<0.01
2-2.9 mg/dL	100 (50.76%)	73 (57.03%)	**0.13**	56 (54.37%)	56 (54.37%)	<0.01
≥ 3 mg/dL	67 (34.01%)	35 (27.34%)	**0.14**	30 (29.13%)	30 (29.13%)	<0.01
INR in Plasma or Blood	1.28 ± 0.3	1.31 ± 0.26	0.08	1.27 ± 0.25	1.32 ± 0.28	**0.17**
INR Lab n	186	122		97	97	
0-1.6 {INR}	186 (94.42%)	122 (95.31%)	0.04	97 (94.18%)	97 (94.18%)	<0.01

1.7-2.2 {INR}	41 (20.81%)	25 (19.53%)	0.03	18 (17.48%)	19 (18.45%)	0.03
≥2.3 {INR}	23 (11.68%)	≤10 (7.81%)	**0.13**	≤10 (9.71%)	≤10 (9.71%)	<0.01
BMI kg/m[2]	28.78 ± 5.61	28.92 ± 5.85	0.02	28.3 ± 4.9	28.85 ± 6.09	0.10
BMI lab n	174	121		92	96	
At least 30 kg/m[2]	114 (57.87%)	77 (60.16%)	0.05	60 (58.25%)	58 (56.31%)	0.04
Alpha-1-Fetoprotein [Mass/volume] in Serum, Plasma or Blood[4]	2564.68 ± 8399.49	2816.82 ± 9560.55	0.03	3180.82 ± 9569.97	3048.04 ± 10283.69	0.01
Alpha-1-Fetoprotein Lab n	106	67		62	54	
**Comorbidities**						
Viral hepatitis	105 (53.3%)	64 (50%)	0.07	57 (55.34%)	57 (55.34%)	<0.01
Ascites	103 (52.28%)	81 (63.28%)	**0.22**	60 (58.25%)	60 (58.25%)	<0.01
Esophageal varices	197 (100%)	128 (100%)		103 (100%)	103 (100%)	
Alcohol related disorders	81 (41.12%)	46 (35.94%)	**0.11**	39 (37.86%)	36 (34.95%)	0.06
Portal vein thrombosis	63 (31.98%)	40 (31.25%)	0.02	31 (30.1%)	33 (32.04%)	0.04
Malignant neoplasm of extrahepatic bile duct	0 (0%)	0 (0%)		0 (0%)	0 (0%)	
**Secondary Malignancy**						
Secondary and unspecified malignant neoplasm of lymph nodes (C77)	15 (7.61%)	10 (7.81%)	0.01	≤10 (9.71%)	≤10 (9.71%)	<0.01
Secondary malignant neoplasm of lung (C78.0)	≤10 (5.08%)	≤10 (7.81%)	**0.11**	≤10 (9.71%)	≤10 (9.71%)	<0.01

[1] Mean + SD; n (%); [2] Cell counts ≤10 are reported as ≤10 in accordance with TriNetX privacy protection policies; totals may not equal 100% and may exceed 100% due to small cell counts and category omission; [3] Standardized mean differences (SMDs) are presented; bold values of > 0.1 indicate imbalance between cohorts; [4]Platelets and Alpha-1-Fetoprotein not included in propsenity score matching.

Table S4. Baseline characteristics of Viral Hepatitis HCC patients treated with Atezolizumab plus Bevacizumab (A + B) or Durvalumab plus Tremelimumab (D + T), before and after propensity score matching.

	Before Matching			After Matching		
**Baseline characteristic**	**Viral Hepatitis A+B** N =559[1,2]	**Viral Hepatitis D+T** N =175[1,2]	**SMD**[3]	**Viral Hepatitis A+B** N = 161[1,2]	**Viral Hepatitis D+T** N =161[1,2]	**SMD**[3]
Age at index	64.98 ± 8.3	65.79 ± 8.34	0.10	65.6 ± 7.6	65.81 ± 8.55	0.03
Sex						
Female	101 (18.07%)	31 (17.71%)	0.01	17 (10.56%)	29 (18.01%)	**0.21**
Male	458 (81.93%)	144 (82.29%)	0.01	144 (89.44%)	132 (81.99%)	**0.21**
Race						
American Indian or Alaska Native	≤10 (1.79%)	≤10 (5.71%)	**0.21**	≤10 (6.21%)	≤10 (6.21%)	<0.01
Asian	113 (20.22%)	20 (11.43%)	**0.24**	17 (10.56%)	20 (12.42%)	0.06
Black or African American	117 (20.93%)	41 (23.43%)	0.06	40 (24.85%)	38 (23.6%)	0.03
Native Hawaiian or Other Pacific Islander	≤10 (1.79%)	≤10 (5.71%)	**0.21**	≤10 (6.21%)	≤10 (6.21%)	<0.01
Other Race	19 (3.4%)	11 (6.29%)	**0.13**	11 (6.83%)	≤10 (6.21%)	0.03
Unknown Race	54 (9.66%)	≤10 (5.71%)	**0.15**	≤10 (6.21%)	≤10 (6.21%)	<0.01
White	246 (44.01%)	95 (54.29%)	**0.21**	89 (55.28%)	89 (55.28%)	<0.01
Ethnicity						
Hispanic or Latino	36 (6.44%)	12 (6.86%)	0.02	≤10 (6.21%)	≤10 (6.21%)	<0.01
Not Hispanic or Latino	388 (69.41%)	125 (71.43%)	0.04	123 (76.4%)	116 (72.05%)	**0.10**
Unknown Ethnicity	135 (24.15%)	38 (21.71%)	0.06	29 (18.01%)	35 (21.74%)	0.09
Labs						
Platelets [#/volume] in Blood[4]	198.19 ± 110.14	206.91 ± 129.7	0.07	205.06 ± 132.53	205.47 ± 130.81	<0.01
Platelet Lab n	542	175		159	161	
Albumin [Mass/volume] in Serum, Plasma or Blood	3.66 ± 0.59	3.51 ± 0.59	**0.26**	3.62 ± 0.6	3.54 ± 0.56	**0.13**
Albumin Lab n	531	172		158	158	
0-2.7 g/dL	162 (28.98%)	76 (43.43%)	**0.30**	62 (38.51%)	66 (40.99%)	0.05
2.8-3.4g/dL	381 (68.16%)	136 (77.71%)	**0.22**	121 (75.16%)	124 (77.02%)	0.04
≥ 3.5 g/dL	485 (86.76%)	159 (90.86%)	**0.13**	148 (91.93%)	145 (90.06%)	0.07
Bilirubin.total [Mass/volume] in Serum, Plasma or Blood	1.1 ± 1.03	1.29 ± 1.52	**0.15**	1.17 ± 1.09	1.3 ± 1.57	0.09
Bilirubin Lab n	534	169		158	156	
0-1.9 mg/dL	526 (94.1%)	165 (94.29%)	0.01	154 (95.65%)	153 (95.03%)	0.03
2-2.9 mg/dL	159 (28.44%)	64 (36.57%)	**0.17**	48 (29.81%)	56 (34.78%)	**0.11**
≥ 3 mg/dL	69 (12.34%)	33 (18.86%)	**0.18**	26 (16.15%)	29 (18.01%)	0.05
INR in Plasma or Blood	1.16 ± 0.24	1.19 ± 0.19	**0.15**	1.16 ± 0.18	1.18 ± 0.17	**0.10**
INR Lab n	516	166		152	152	

0-1.6 {INR}	515 (92.13%)	166 (94.86%)	**0.11**	152 (94.41%)	152 (94.41%)	<0.01
1.7-2.2 {INR}	32 (5.73%)	17 (9.71%)	**0.15**	13 (8.08%)	14 (8.7%)	0.02
≥2.3 {INR}	19 (3.4%)	10 (5.71%)	0.11	10 (6.21%)	10 (6.21%)	<0.01
BMI kg/m²	26.24 ± 5.15	26.04 ± 5.16	0.04	25.91 ± 5.32	26.18 ± 5.22	0.05
BMI lab n	480	163		146	149	
At least 30 kg/m²	201 (35.96%)	68 (38.86%)	0.06	64 (39.75%)	63 (39.13%)	0.01
Alpha-1-Fetoprotein [Mass/volume] in Serum, Plasma or Blood[4]	5163.52 ± 15193.59	3135.81 ± 10279.27	0.16	4126.94 ± 13876.64	3381.41 ± 10649.13	0.06
Alpha-1-Fetoprotein Lab n	268	81		71	75	
**Comorbidities**						
Viral hepatitis	559 (100%)	175 (100%)		161 (100%)	161 (100%)	
Ascites	101 (18.07%)	72 (41.14%)	0.52	52 (32.3%)	58 (36.03%)	0.08
Esophageal varices	127 (22.72%)	69 (39.43%)	0.37	60 (37.27%)	57 (35.4%)	0.04
Alcohol related disorders	146 (26.12%)	64 (36.57%)	0.23	53 (32.92%)	56 (34.78%)	0.04
Portal vein thrombosis	92 (16.46%)	40 (22.86%)	0.16	35 (21.74%)	36 (22.36%)	0.01
Malignant neoplasm of extrahepatic bile duct	≤10 (1.79%)	0 (0%)	0.19	0 (0%)	0 (0%)	
**Secondary Malignancy**						
Secondary and unspecified malignant neoplasm of lymph nodes (C77)	38 (6.8%)	≤10 (5.71%)	0.04	13 (8.08%)	≤10 (6.21%)	0.07
Secondary malignant neoplasm of lung (C78.0)	36 (6.44%)	≤10 (5.71%)	0.03	10 (6.21%)	≤10 (6.21%)	<0.01

[1] Mean + SD; n (%); [2] Cell counts ≤10 are reported as ≤10 in accordance with TriNetX privacy protection policies; totals may not equal 100% and may exceed 100% due to small cell counts and category omission; [3] Standardized mean differences (SMDs) are presented; bold values of > 0.1 indicate imbalance between cohorts; [4]Platelets and Alpha-1-Fetoprotein not included in propsenity score matching.
